# Invasive Pneumococcal Disease in Refugee Children, Germany

**DOI:** 10.3201/eid2410.180253

**Published:** 2018-10

**Authors:** Stephanie Perniciaro, Matthias Imöhl, Mark van der Linden

**Affiliations:** University Hospital RWTH Aachen, Aachen, Germany

**Keywords:** invasive pneumococcal disease, refugees, asylum seekers, pneumococcal conjugate vaccine, refugee health, vaccines, bacteria, Germany

## Abstract

Refugee children in Germany are not routinely given a pneumococcal conjugate vaccine. Cases of invasive pneumococcal disease (IPD) in 21 refugee children were compared with those in 405 Germany-born children for 3 pneumococcal seasons. Refugee children had significantly higher odds of vaccine-type IPD and multidrug-resistant IPD than did Germany-born children.

Germany has taken in >1 million refugees since 2015 (*1*), more than one third of whom were children <18 years of age (*2*). Invasive pneumococcal disease (IPD) is a major cause of childhood death, especially in resource-poor environments (*3*). Conflict settings are associated with outbreaks of vaccine-preventable diseases for reasons ranging from poor sanitation in refugee holding areas to the rapid movement of refugees, which, in turn, allows for a similarly rapid spread of disease and the interruption of immunization services because of the lack of personnel (*4*).

Of the 10 most frequent countries of origin for refugees arriving in Germany in 2017 (Syria, Iraq, Afghanistan, Turkey, Iran, Nigeria, Eritrea, Russia, Somalia, and Albania), 6 have a national vaccination program that includes pneumococcal conjugate vaccines (PCVs) (*5*); however, because of the crisis conditions facing those who fled, timely infant vaccination is unlikely (*4*). Vaccine-preventable disease outbreaks have been reported in refugee housing facilities in Germany (*6*,*7*), and most of these cases have originated after arrival in Germany. The vaccination program for newly arrived refugees does not include PCVs (*8*).

Since 1997, the German National Reference Center for Streptococci (GNRCS) has been collecting bacterial isolates from IPD cases in children occurring throughout Germany. We compared IPD isolates received from known refugee children residing in Germany to IPD isolates from Germany-born children for the 2014–15, 2015–16, and 2016–17 pneumococcal seasons.

## The Study

In this retrospective, unmatched case-control study, we considered all 514 isolates from children (<16 years of age) with IPD in the GNRCS collection isolated during July 1, 2014–June 30, 2017, for inclusion in the analysis. We defined a case of IPD as *Streptococcus pneumoniae* (identified by optochin sensitivity and bile solubility) isolated from a normally sterile site. For the analysis, case isolates were from refugee children with IPD, and control isolates were from Germany-born children with IPD. Refugee status was documented by GNRCS personnel in conjunction with determining vaccination status, as described elsewhere (*9*). Because identification of refugee status was tied to the determination of vaccination status, all children with an unknown vaccination status were excluded.

We determined serotype by using Neufeld’s Quellung reaction and antimicrobial drug resistance by MIC testing, as previously described (*10*). Antimicrobial drug resistance was defined by the Clinical and Laboratory Standards Institute 2015 breakpoints (*11*).

We calculated odds ratios (ORs) and 95% CIs with R software version 3.4.0 (https://www.r-project.org/foundation) using Firth’s bias-reduced logistic regression and adjusted for age and sex using the logistf package (*12*). We assigned statistical significance to ORs for which the 95% CI did not exceed 1. Variables considered in the analysis were age, sex, vaccination status, and refugee status of the patient, as well as the serotype and antimicrobial drug resistance profile of the isolate.

During July 1, 2014–June 30, 2017, the GNRCS received 21 IPD isolates from known refugee children (Table). The average age of infection in refugee children was 3 years. Of these cases, 11 (44%) had an unknown clinical diagnosis (6 isolates were from blood, 2 from cerebrospinal fluid, 3 from other exudates), 4 (19%) were from meningitis, 3 (14%) were from sepsis, and 3 (14%) were from pneumonia. Thirteen refugee children, all of whom were unvaccinated, had vaccine-type IPD (62% overall; 2014–15, 67%; 2015–16, 67%; 2016–17, 50%). Only 2 refugee children, both with non–vaccine-type IPD, had been vaccinated with 13-valent PCV, both with only 1 dose, after arrival in Germany.

**Table:** Invasive pneumococcal disease in refugee children and German-born children, July 1, 2014–June 30, 2017*

IPD season	No. patients	Mean patient age, y	No. (%) patients				
			Unvaccinated	PCV13 vaccinated	VT serotype	Non-VT serotype	Resistant to >3 classes of antimicrobial drugs
Refugee children in Germany							
2014–15	3	1	3 (100)	0	2 (67)	1 (33)	1 (33)
2015–16	12	3	10 (83)	2 (17)	8 (67)	4 (33)	4 (33)
2016–17	6	5	5 (83)	0	3 (50)	3 (50)	3 (50)
Total	21	3	18 (86)	2 (9)	13 (62)	8 (38)	8 (38)
Germany-born children							
2014–15	107	2	19 (18)	73 (68)	28 (26)	79 (74)	5 (5)
2015–16	122	2	21 (17)	86 (70)	19 (16)	103 (84)	1 (0.8)
2016–17	176	3	45 (26)	117 (66)	28 (16)	148 (84)	4 (2)
Total	405	2	85 (21)	276 (68)	75 (19)	330 (81)	10 (2)

*VT serotypes are 4, 6B, 9V, 14, 18C, 19F, 23F, 1, 5, 7F, 3, 6A, and 19A. Antimicrobial drug resistance is determined according to the 2015 breakpoints of the Clinical and Laboratory Standards Institute (*11*). IPD, invasive pneumococcal disease; PCV13, 13-valent pneumococcal conjugate vaccine; VT, vaccine type.

We determined vaccination status for 405 isolates from Germany-born children (Table). The average age at the time of infection was 2 years. Fifty-four children (13%) had pneumonia, 88 (22%) had sepsis, 130 (32%) had meningitis, and 133 cases (33%) had an unknown or other diagnosis. Seventy-five cases in this group were vaccine-type IPD (19% overall; 2014–15, 26%; 2015–16, 16%; 2016–17, 16%). Refugee children had significantly higher odds of contracting vaccine-type IPD (OR 6.60, 95% CI 2.73–16.84) over the study period.

Eight isolates (38% overall; 2014–15, 33%; 2015–16, 33%; 2016–17, 50%) from refugee children were resistant to >3 classes of antimicrobial drugs, compared with 10 isolates (2% overall; 2014–15, 4%; 2015–16, 1%; 2016–17, 2%) from Germany-born children. Refugee children had significantly higher odds (OR 23.84, 95% CI 7.98–72.73) of contracting antimicrobial-resistant IPD over the study period. Five vaccine-type isolates (38% overall; 2014–15, 50%; 2015–16, 25%; 2016–17, 67%) from refugees were resistant to >3 of antimicrobial drugs, compared with 5 vaccine-type isolates (7% overall; 2014–15, 11%; 2015–16, 5%; 2016–17, 4%) from Germany-born children. Among vaccine-type IPD cases, refugee children were significantly more likely (OR 8.82, 95% CI 2.13–40.10) to have antimicrobial drug–resistant infections.

IPD incidence estimates are shown in Technical Appendix Table 1). For single-season ORs, the CIs were often wide, and the sample sizes in refugee children were low (2014–15, n = 3; 2015–16, n = 12; 2016–17, n = 6). These ORs are shown in Technical Appendix Table 2 and should be interpreted cautiously.

## Conclusions

Refugee children in Germany are at greater risk of contracting vaccine-type IPD, antimicrobial drug–resistant IPD, and antibiotic-resistant vaccine-type IPD. As such, a PCV program for refugee children may be worth considering in Germany. Vaccination in newly arrived refugees presents an opportunity to cost-effectively, safely, and humanely protect a vulnerable population from negative health outcomes resulting from vaccine-preventable diseases (*13*). Given that children in Germany with insecure residence status are twice as likely to be incompletely vaccinated (*2*), a PCV program for refugee children in Germany might require additional follow-up measures to ensure consistency and provide sufficient protection, particularly because PCV dosing in Germany-born children with IPD has been lax (*9*). A PCV program could help reduce antimicrobial drug–resistant pneumococcal infections, the carriage of resistant strains (*14*), overall antimicrobial drug use, and the prevalence of resistance genes within the pneumococcal population (*15*).

The IPD case numbers from refugees are low, but the proportion of vaccine-type isolates and antimicrobial drug–resistant isolates from refugee children are nevertheless much higher than those of Germany-born children. Children of unknown vaccination status (n = 109) were excluded from the analysis; because of the small sample sizes, if even 1 additional child with vaccine-type IPD per year was a refugee, the effects we describe would be magnified (Technical Appendix Table 3).

The risk of vaccine-type IPD is low among fully vaccinated children in Germany. However, among unvaccinated and undervaccinated children, a reintroduction of vaccine-type pneumococci may result in increased risk of pneumococcal disease. Without intervention, refugee children may continue to constitute a special risk group for vaccine-type IPD and antimicrobial drug–resistant IPD in Germany. Fully immunizing these children against vaccine-type IPDs may help reduce the risk for IPD illness and death in Germany.

Technical Appendix Discussion of the estimated incidence of invasive pneumococcal disease in refugee and Germany-born children.
