# Supplementary material for: Invasive Pneumococcal Disease in Refugee Children, Germany
Source: Emerg Infect Dis. 2018 Oct;24(10):1934–6. doi: 10.3201/eid2410.180253 (PMC6154141; doi:10.3201/eid2410.180253)
Supplement: Technical Appendix — Discussion of the estimated incidence of invasive pneumococcal disease in refugee and Germany-born children. [file 18-0253-Techapp-s1.pdf]

Article DOI: https://doi.org/10.3201/eid2410.180253

Invasive Pneumococcal Disease in Refugee Children, Germany

Technical Appendix

The estimated incidence of invasive pneumococcal disease (IPD) per 100,000 children is shown in Technical Appendix Table 1. The odds ratios (OR) and 95% confidence intervals (CI) for bias-reduced, multivariate logistic regression models are shown in Technical Appendix Table 2, describing vaccine-type IPD, multiple–drug-resistant IPD (resistance to ≥3 classes of antimicrobial drugs) and multiple drug resistance in vaccine-type IPD, adjusted for age and sex. These models compare refugee children with German-born children for July 1, 2014–June 30, 2017. The values for the single-year comparisons correspond to cohorts with small sample sizes and therefore should be interpreted cautiously. Because of the small sample sizes, we have also shown a model sensitivity assessment for vaccine-type IPD in Technical Appendix Table 3, showing the magnified effect of adding only 1 additional vaccine-type IPD case to the refugee children group per season over the study period. Technical Appendix Table 3 is for reference only and does not represent the actual findings of this study.

Technical Appendix Table 1. Estimated incidence of invasive pneumococcal disease (IPD) in refugee children and German-born children <16 years of age, July 1, 2014–June 30, 2017

Category	N	Estimated population	Estimated IPD incidence per 100,000 population
Refugee children*	21	414,088	5.07
German-born children†	405	11,823,429	3.43

*Population estimates for refugee children from the Federal Office for Migration and Refugees, Asylum Seekers Report, 2015, 2016, 2017.
†Population estimates for German-born children from the Federal Statistical Office, 2018.

Technical Appendix Table 2. Age- and sex-adjusted odds ratios for the study period, July 1, 2014–June 30, 2017*

IPD class	OR	95% CI
VT IPD		
Overall	6.60	2.73–16.84
2014–15	7.69	0.78–106.40
2015–16	9.08	2.75–33.99
2016–17	4.82	0.99–23.47
MDR IPD		
Overall	23.84	7.98– 72.73
2014–15	29.66	1.33–4612.47
2015–16	98.54	8.56–7448.90
2016–17	28.09	4.90–168.15
VT MDR IPD		
Overall	8.82	2.13–40.10
2014–15	6.17	0.19–1011.13
2015–16	42.30	1.30 to 1.96×10^7
2016–17	22.71	2.07–441.76

*CI, confidence interval; IPD, invasive pneumococcal disease; MDR, multidrug-resistant; OR, odds ratio; VT, vaccine-type.

Technical Appendix Table 3. Bias-reduced logistic regression model sensitivity assessment*

VT IPD	OR	95% CI
Overall	8.06	3.46–20.09
2014–15	10.44	1.24–140.65
2015–16	10.05	3.12–37.12
2016–17	6.07	1.42–28.10

*CI, confidence interval; IPD, invasive pneumococcal disease; OR, odds ratio; VT, vaccine-type
